# A Reinforcement Learning-Based Generative Approach for Event Temporal Relation Extraction

**DOI:** 10.3390/e27030284

**Published:** 2025-03-09

**Authors:** Zhonghua Wu, Wenzhong Yang, Meng Zhang, Fuyuan Wei, Xinfang Liu

**Affiliations:** 1 School of Computer Science and Technology, Xinjiang University, Urumqi 830017, China; wuzhonghua@stu.xju.edu.cn (Z.W.); menka@stu.xju.edu.cn (M.Z.); wfy@stu.xju.edu.cn (F.W.); xmta1724@stu.xju.edu.cn (X.L.); 2 Xinjiang Key Laboratory of Multilingual Information Technology, Xinjiang University, Urumqi 830017, China

**Keywords:** temporal relation extraction, generative models, multi-task learning, dependency path, reinforcement learning, policy gradient method

## Abstract

Event temporal relation extraction is a crucial task in natural language processing, aimed at recognizing the temporal relations between event triggers in a text. Despite extensive efforts in this area, the existing methods face two main issues. Firstly, the previous models for event temporal relation extraction mainly rely on a classification framework, which fails to output the crucial contextual words necessary for predicting the temporal relations between two event triggers. Secondly, the prior research that formulated natural language processing tasks as text generation problems usually trained the generative models by maximum likelihood estimation. However, this approach encounters potential difficulties when the optimization objective is misaligned with the task performance metrics. To resolve these limitations, we introduce a reinforcement learning-based generative framework for event temporal relation extraction. Specifically, to output the important contextual words from the input sentence for temporal relation identification, we introduce dependency path generation as an auxiliary task to complement event temporal relation extraction. This task is solved alongside temporal relation prediction to enhance model performance. To achieve this, we reformulate the event temporal relation extraction task as a text generation problem, aiming to generate both event temporal relation labels and dependency path words based on the input sentence. To bridge the gap between the optimization objective and task performance metrics, we employ the REINFORCE algorithm to optimize our generative model, designing a novel reward function to simultaneously capture the accuracy of temporal prediction and the quality of generation. Lastly, to mitigate the high variance issue encountered when using the REINFORCE algorithm in multi-task generative model training, we propose a baseline policy gradient algorithm to improve the stability and efficiency of the training process. Experimental results on two widely used datasets, MATRES and TB-DENSE, show that our approach exhibits competitive performance.

## 1. Introduction

Event temporal relation extraction is a challenging and significant task in the field of natural language processing, as it contributes to the research and development of many downstream tasks such as reading comprehension [1,2], question answering [3,4], text summarization [5,6], and future event prediction [7,8]. Event temporal relation extraction aims to automatically extract the temporal relationship between a given pair of events and further construct a temporal graph. Events are typically expressed by event triggers, which are usually single or consecutive verbs within event sentences. While event triggers can often be detected effectively, extracting relations between events, especially temporal relations, remains a challenging task.

Existing methods for event temporal relation extraction are primarily based on pre-trained language models to capture the representations of event mentions, with various learning and reasoning approaches for further improvements. To enhance the quality of event representations, Trong et al. [9] employed reinforcement learning to select the optimal contextual sentences, while Mathur et al. [10] introduced rhetorical discourse features and temporal parameters, achieving state-of-the-art performance. Additionally, other studies [11,12] utilized graph neural networks to avoid complex feature engineering. From a learning perspective, some research [13,14] enriched models through auxiliary training tasks to provide supplementary supervision signals, while others [15,16] leveraged heuristic cues and patterns to introduce distant supervision. Despite some progress, the existing methods still face two main issues.

Firstly, the previous work has primarily treated event temporal relation extraction as a multiclass classification problem, where the model’s sole output is merely a temporal relation label. A key challenge with this classification approach is that the current temporal relation extraction studies are unable to generate the crucial contextual information necessary for identifying the temporal relation between event triggers. Additionally, this approach fails to fully utilize the semantic information of the labels, as they are treated merely as numerical indices during training [17,18]. In this study, the important context consists of words in the input sentence that help to uncover the temporal relations between the given event triggers. This limitation in the current event temporal relation extraction methods is undesirable because we believe that incorporating important contextual information as part of the model’s output can provide richer training signals. Therefore, we need to explore a new strategy that integrates important task-relevant context into the model’s output to aid the model training process.

Secondly, recent studies [19,20,21] have demonstrated that it is possible to restructure natural language processing tasks as text generation problems, where generative models are typically trained by maximum likelihood estimation. However, this approach encounters a misalignment between the optimization goal (i.e., maximizing likelihood) and the performance metric of the task (i.e., the accuracy of relation prediction). Moreover, the number of words present in the dependency path may surpass that of the temporal labels in the output sequence. As a result, when training with likelihood maximization, the role of temporal labels as learning signals is weakened within a multi-task learning framework. Therefore, we need to explore a new algorithm for training generative models. This method should allow us to directly use task performance metrics as rewards for training the generative model, while also providing the flexibility to adjust the weighting between temporal relation labels and important contextual words during the training process.

In this study, we introduce a novel generative method for event temporal relation extraction (GenTRE) and adopt an innovative dependency path generation and reinforcement learning algorithm to address the two aforementioned issues. Firstly, to output the important contextual words necessary for predicting the temporal relations between two event mentions from an input sentence, we introduce a multi-task learning technique that allows for the simultaneous generation of both temporal relations and important contextual words. Inspired by relation extraction models in information extraction [22,23], we consider the words along the dependency path linking two event triggers in the dependency tree to be crucial contextual words. We consider dependency path generation to be a crucial task related to temporal label prediction, and training a model to jointly generate temporal relation labels and dependency path words can improve performance. To achieve this, we reformulate the event temporal relation extraction task through a novel generative approach, aiming to generate both temporal relation labels and the dependency path words between event triggers. Our model integrates temporal relation labels and dependency path words into a unified output sequence, which is generated in an autoregressive manner based on the input sentence. To solve this sequence-to-sequence challenge, we leverage the generative pre-trained language model T5 [24] to produce the complete output sequence.

Secondly, to resolve the discrepancy between the optimization objective and the performance metrics of the task, we propose using the policy gradient method REINFORCE [25] to train our generative model, allowing the task performance metrics to be directly used as rewards for training the model. Specifically, our training reward consists of two independent components—the accuracy of the predicted temporal labels and the similarity between the generated output sequence and the gold output sequence. This setup enables a reasonable allocation of importance between the temporal relation labels and the relevant contextual words during training. Additionally, inspired by studies [9,26], we introduce a novel auxiliary reward that encourages similarity in temporal relation prediction ability between the predicted sentence and the input sentence, thereby enriching the training signals.

Finally, due to the high variance in gradient estimates when training generative models with the REINFORCE algorithm, which leads to instability in the model training process and susceptibility to issues such as vanishing or exploding gradients, we introduce a baseline policy gradient approach to train the generative model. Inspired by the previous research [27,28], our method aims to enhance the training process and ensure a more reliable model performance. Unlike traditional policy gradient methods, this approach utilizes a value function-based baseline to decrease the variance of the policy gradients, thereby improving the stability and efficiency of the algorithm.

In the experiments, we first evaluated our model on two benchmark datasets, MATRES and TB-DENSE, demonstrating that our model exhibits a competitive and robust performance. Additionally, we conducted a series of comprehensive evaluations, including the impact of training data size, the influence of reward function weights, and the effectiveness of the baseline policy. The experimental results indicate that our method not only enhances the stability and efficiency of the model but also improves its robustness and generalization ability.

In summary, our contributions are as follows: (1) We propose a novel generative model for event temporal relation extraction, where the task is modeled as a sequence-to-sequence generation problem. Compared to classification-based methods, the generative approach has the capability to encode label semantic information within the target sequence. (2) We introduce a dependency-based method for temporal relation extraction, incorporating dependency path generation as an auxiliary task. This approach helps the model better perceive the fundamentally associated knowledge between two event triggers. (3) We develop a baseline policy gradient algorithm to effectively mitigate the high variance issue and train the generative model. Additionally, we design a new reward function to balance the importance of temporal relation label prediction and contextual word extraction. (4) We conduct extensive experiments on two benchmark datasets, MATRES and TB-DENSE, demonstrating that our model exhibits a competitive and robust performance.

## 2. Related Work

### 2.1. Event Temporal Relation Extraction

Early research on event temporal relation extraction primarily modeled it as a pairwise classification problem and used statistical machine learning techniques and handcrafted features to extract the temporal relations [29,30]. With the rapid development of deep learning, convolutional neural networks and recurrent neural network models were subsequently introduced. Some researchers applied these models to identify temporal relations [31,32,33], achieving notable improvements. In recent years, with the advent of large-scale pre-trained language models, some studies [34,35,36,37] have significantly enhanced the performance of event temporal relation extraction by leveraging the contextual representations learned from these models. These models have been shown to automatically extract reliable event features for temporal relation extraction when provided with high-quality data, thereby significantly reducing the manual effort required from engineers.

Overall, research on event temporal relation extraction has demonstrated a variety of methods and techniques, highlighting the importance of considering contextual embeddings, structured learning frameworks, and hyperbolic geometry spaces for this task. Despite significant efforts, existing methods typically approach event temporal relation extraction as a classification problem, which cannot output the crucial background information necessary for predicting the temporal relations between two event triggers. To address the limitations of the previous methods, we introduce a multi-task learning technique that incorporates dependency path generation as an auxiliary task for temporal relation prediction, as well as reformulate event temporal relation extraction as a text generation problem, aiming to simultaneously generate temporal relation labels and dependency path words from the input sentence. As far as we know, we are the first to model event temporal relation extraction using generative models and achieve favorable results.

### 2.2. Generation-Based Information Extraction

In recent years, an increasing number of studies have focused on adopting new generative paradigms to address information extraction tasks, which include (but are not limited to) traditional discriminative tasks such as classification and structured prediction. Zhang et al. [18] and Paolini et al. [19] transformed information extraction tasks into text translation tasks with label augmentation. Additionally, some studies [38,39,40] have designed linearization schemes with constrained decoding strategies, while others [41,42,43] have employed template-based conditional generation. Although this paradigm may seem straightforward, generative methods have reported competitive results in sentence-level benchmarks.

However, the previous studies have not explored the use of generative models for event temporal relation extraction. Furthermore, these approaches typically trained generative models by maximizing the likelihood of the gold output sequence, which created a discrepancy between the optimization goal and the performance metrics of the task. In this work, we reframe event temporal relation extraction as a text generation problems and introduce a baseline policy gradient method to tackle the issue, where maximizing the likelihood function in generative models may reduce the significance of temporal labels as training signals in a multi-task learning setup. This method further enhances the stability and convergence speed of the generative model. As far as we are aware, this is the first study to apply a baseline policy gradient algorithm to event temporal relation extraction.

## 3. Methods

**Figure 1:**
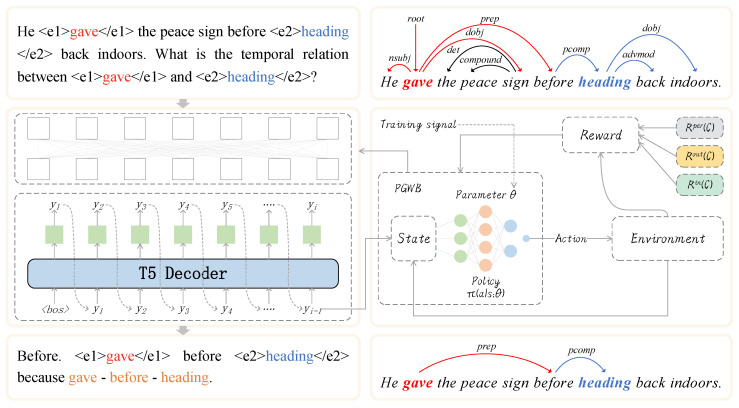
The architecture of the proposed generative method for event temporal relation extraction. Red text indicates the head event, blue text the tail event, and yellow text the words in the shortest dependency path. Red/blue arrows denote the dependency relations of the head/tail events.

In this study, we introduce a generative model for event temporal relation extraction, treating the task as a sequence-to-sequence text generation problem. For each event pair in a sentence, we predict the type of temporal relation it belongs to. Figure 1 provides an overview of our method, which consists of the following three main components: (1) Sequence-to-Sequence Modeling: We propose a dependency-based generative method for event temporal relation extraction to simultaneously generate temporal relation labels and dependency path words from the input sentence. (2) Maximum Likelihood Pre-training: We first pre-train the generative model using maximum likelihood estimation to enable it to produce output sequences that align more closely with the training data distribution. (3) Baseline Policy Gradient Algorithm: We introduce a baseline policy gradient algorithm to resolve the discrepancy between the optimization objective and the performance metrics of the task, as well as to improve the stability and convergence speed of the generative model. Each component will be described in detail below.

### 3.1. Sequence-to-Sequence Modeling

For a sentence *W* with *n* tokens $w_{1},w_{2},\ldots ,w_{n}$, which can have multiple event mentions $\left\{e_{1},e_{2},\ldots ,e_{m}\right\}$, the goal of event temporal relation extraction is to predict the temporal relation type between event pairs $\left(e_{s},e_{t}\right)$. In this work, we shift from traditional classification methods [37,44,45] to a generative approach. Our generative method adopts a sequence-to-sequence architecture, with the input consisting of the sentence *W* and the two event triggers, $e_{s}$ and $e_{t}$. The model generates an output sequence that consists of temporal labels and the dependency path linking $e_{s}$ and $e_{t}$ in *W*’s dependency tree, thus facilitating multi-task learning by generating crucial contextual words.

Specifically, the input sequence *I* of our generative event temporal relation extraction model is obtained by combining the input sentence *W* and the prompt $P\left(e_{s},e_{t}\right)$, as follows:

(1) $$I=W:P\left(e_{s},e_{t}\right)$$

where $P\left(e_{s},e_{t}\right)$ is used to specify the temporal relation prediction task for $e_{s}$ and $e_{t}$. In this study, we define $P\left(e_{s},e_{t}\right)$ with a straightforward template structured as “What is the temporal relationship between $e_{s}$ and $e_{t}$?” Furthermore, the output sequence *O* in the task of generative event temporal relation extraction is concatenated in the following way:

(2) $$O=L:D\left(e_{s},e_{t}\right)$$

where *L* indicates the type of temporal relation (also known as the temporal relation label) between the two event triggers, $e_{s}$ and $e_{t}$. Additionally, $D\left(e_{s},e_{t}\right)$ refers to the dependency path connecting $e_{s}$ and $e_{t}$ within the sentence *W*. The following provides the input and output sequences for the given example:

- Input: He gave the peace sign before heading back indoors. What is the temporal relation between gave and heading?
- Output: Before. gave happened before heading because gave before heading.

In this work, we only consider the event pairs from adjacent sentences because if we also include the event pairs from non-adjacent sentences, the shortest dependency path becomes overly complex, which is beyond the scope of this study. When two events are located in different sentences, a key challenge is how to represent the cross-sentence dependency path. In this paper, we adopt the hypothesis proposed by Cheng et al. [46], which suggests that two adjacent sentences share a “common root” node. As a result, the cross-sentence dependency path can be depicted as two shortest branches of the dependency path, each extending from the endpoints to the “common root” node.

Using this approach, our generative event temporal relation extraction model can be trained simultaneously for two highly related tasks—temporal relation prediction and dependency path generation. This enables multi-task training, which enhances the model’s performance. In the training process, for each transformed input–output pair (I,O) in the data, the sequence-to-sequence problem is addressed by using a pre-trained T5 encoder–decoder model. Specifically, the T5 model is trained on these transformed pairs (I,O) from the training data. During inference, given an input sentence and two event mentions, the trained T5 model generates an output sequence. The first token from this sequence is then extracted to predict the temporal relation label.

To summarize, we introduce dependency path generation as an auxiliary task for event temporal relation extraction, allowing the concurrent generation of both temporal relation labels and the crucial context. This multi-task learning strategy effectively improves the accuracy of temporal relation prediction.

### 3.2. Maximum Likelihood Pre-Training

Before training the baseline policy gradient algorithm, we first train T5 using a maximum likelihood objective to guide it in generating text over the transformed input–output pairs (I,O). This approach effectively limits the vast action space involved in text generation, improving the learning efficiency of the baseline policy gradient algorithm [47,48]. In other words, by pre-training T5 with maximum likelihood, we can better adapt it to the event temporal relation extraction task. Then, we integrate the baseline policy gradient algorithm with T5 to conduct joint training for the temporal relation extraction task.

In this study, we employ cross-entropy loss for the pre-training of the T5 model, which is a generative model primarily focused on mapping input sequences to their corresponding output sequences. To enhance the likelihood of each sample, we optimize the model by minimizing the negative log-likelihood of the output sequence *O* conditioned on the input sequence *I*, as follows:

(3) $$L_{\mathrm{gen}\;}=-\log P\left(O|I\right)$$

where the probability P(O∣I) is computed through the distribution returned by the decoder. The objective of the generative loss is to maximize the likelihood that the model produces an output sequence consistent with the distribution of the training data. To achieve the reconstruction, we reverse the process by transforming the generated output sequence *C* back into the input sequence *I*. Then, we calculate the negative log-likelihood of obtaining the input sequence *I* when *C* is provided as the input, as in the following equation:

(4) $$L_{re}=-\log P\left(I|C\right)$$

The reconstruction loss helps to ensure that the model’s generated output sequence closely resembles the original input sequence. To finalize the process, we combine the generative loss and reconstruction loss with specified weights to form the final training loss function, as in the following equation:

(5) $$L_{\mathrm{likelihood}\;}=\alpha_{\mathrm{gen}\;}L_{\mathrm{gen}\;}+\alpha_{\mathrm{re}\;}L_{\mathrm{re}\;}$$

where $\alpha_{\mathrm{gen}}$ and $\alpha_{\mathrm{rec}}$ are the weights for the generative loss and the reconstruction loss, which can be adjusted through hyperparameters.

In conclusion, by pre-training the T5 model using maximum likelihood estimation for the event temporal relation extraction task, it becomes better at producing output sequences that match the distribution of the training data, which ultimately enhances the performance of the baseline policy gradient algorithm.

### 3.3. Baseline Policy Gradient Algorithm

As mentioned in the introduction, we utilize a baseline policy gradient algorithm to train our generative event temporal relation extraction model. In this approach, label accuracy is integrated into the reward function, serving as a direct training signal. The model parameters are first initialized using maximum likelihood estimation and, subsequently, they are refined through iterative optimization with the baseline policy gradient algorithm. The baseline policy gradient algorithm’s flexibility enables the inclusion of a term in the reward function that reflects the similarity between the predicted output sequence *C* from T5, the gold output sequence *O*, and the input sequence *I* for the training of our generative model. In contrast to traditional policy gradient algorithms, the baseline policy gradient algorithm incorporates a value function-based baseline, which helps to reduce the variance of the policy gradient thus improving the algorithm’s efficiency and stability. Algorithm 1 presents the implementation details of the baseline policy gradient algorithm. Drawing from this description, we have developed three reward components for the baseline policy gradient algorithm to facilitate the training of the generative model.**Algorithm 1:** Policy gradient method with baseline for estimating *π_θ_* ≈ *π*_∗_
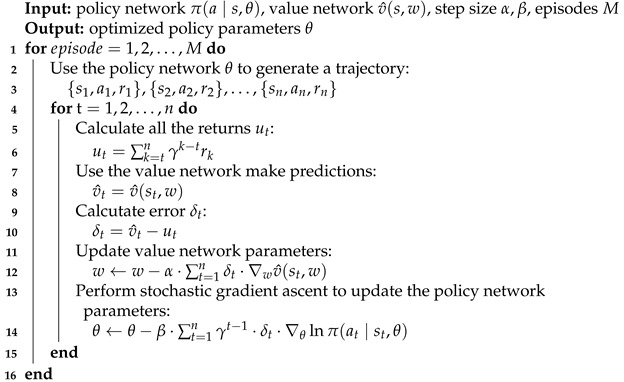


**Accuracy-based Reward $R^{\mathrm{acc}}\left(C\right)$**: This reward is computed by evaluating the accuracy of the temporal relation label *L* within the generated sequence *C*. Specifically, if *L* matches the temporal relation provided between $e_{s}$ and $e_{t}$ in *W*, then $R^{\mathrm{acc}}\left(C\right)=1$; otherwise, it is 0. Thus, the calculation of the accuracy-based reward $R^{\mathrm{acc}}\left(C\right)$ is as follows:

(6) $$R^{\mathrm{acc}}\left(C\right)=\frac{1}{S}\sum_{i=1}^{S}\Phi \left(C_{i}=O_{i}\right)$$

where $C_{i}$ denotes the *i*-th output sequence produced by the model, $O_{i}$ is the corresponding expected output sequence, and *S* refers to the total number of samples. The symbol Φ is used to denote an indicator function, which is 1 if the first token of $C_{i}$ and $O_{i}$ are equal, and 0 otherwise. This reward term aims to encourage the generation of correct temporal labels, thereby improving the overall performance of the model.

**Output-based Reward $R^{\mathrm{out}}\left(C\right)$**: This reward term is determined by evaluating how closely the generated sequence *C* matches the reference output sequence *O*. In particular, we use the ROUGE-2 metric [49] to calculate this reward term, i.e., $R^{\mathrm{out}}\left(C\right)=\mathrm{ROUGE}-2\left(C,O\right)$. Therefore, the calculation of the output-based reward $R^{\mathrm{out}}\left(C\right)$ is as follows:

(7) $$R^{\mathrm{out}\;}\left(C\right)=\frac{1}{S}\sum_{i=1}^{S}\mathrm{ROGUE}-2\left(C_{i},O_{i}\right)$$

where ROUGE-2 is the similarity calculation function. This reward term aims to motivate the generation model to produce temporal dependency paths that closely resemble the desired output sequences, which ultimately enhances the model’s overall performance.

**Input-Based Reward $R^{\mathrm{in}}\left(C\right)$**: Our goal is to generate the dependency path between $e_{s}$ and $e_{t}$ for multi-task learning in event temporal relation extraction. Given that the dependency path is designed to capture key contextual information that reveals the temporal relation in *W*, and considering that the input *I* is tailored for temporal prediction, we posit that *I* and *O* should share a similar meaning. Building on this idea, we propose a novel reward term to enhance the similarity between the generated sequence *C* from T5 and the input sequence *I*. Specifically, we feed both *C* and *I* into T5’s encoder to obtain their respective representation vectors, V(C) and V(I). The reward is then calculated based on the similarity between these representation vectors, i.e., $R^{\mathrm{in}}\left(C\right)=\mathrm{similarity}\left(V\left(C\right),V\left(I\right)\right)$. Therefore, the calculation of the input-based reward $R^{\mathrm{in}}\left(C\right)$ is as follows:

(8) $$R^{\mathrm{in}\;}\left(C\right)=\frac{1}{S}\sum_{i=1}^{S}\mathrm{similarity}\left(C_{i},O_{i}\right)$$

where similarity is a similarity calculation function defined based on the specific task and requirements. Common techniques for assessing text similarity include approaches like cosine similarity and edit distance.

Thus, the overall reward function R(C) for training our generative event temporal relation extraction model is as follows:

(9) $$R\left(C\right)=\alpha_{\mathrm{acc}}R^{\mathrm{acc}}\left(C\right)+\alpha_{\mathrm{out}}R^{\mathrm{out}\;}\left(C\right)+\alpha_{\mathrm{in}}R^{\mathrm{in}\;}\left(C\right)$$

where $\alpha_{\mathrm{acc}},\alpha_{\mathrm{out}}$, and $\alpha_{\mathrm{in}}$ are the weighting parameters. By doing so, we can ensure that label accuracy—our main performance objective—is effectively emphasized, preventing it from being overshadowed by generation rewards during training.

In practice, the baseline policy gradient algorithm utilizes gradient descent to adjust the policy function’s parameters, aiming to maximize its expected reward. Specifically, P(C∣I) represents the distribution of sequences generated by T5. In our approach, we employ the baseline policy gradient algorithm to train T5 by minimizing the negative expected reward R(C) of the sequences it generates, as follows:

(10) $$L_{RL}=-E_{C∼P(C|I)}\left[R\left(C\right)\right]$$

where *C* represents all possible choices by T5. Using the policy gradient method and a single roll-out sampling of the generated sequences *C* [50], the gradient of $L_{RL}$ can be approximated and used for training as follows:

(11) $$\nabla L_{RL}=-\left(R\left(C\right)-base\right)\nabla \log P\left(C|I\right)$$

where base is the baseline used to minimize variance. In this context, the baseline base is derived through the following equation:

(12) $$base=\frac{1}{\left|B\right|}\sum_{k=1}^{\left|B\right|}R\left(C^{k}\right)$$

where |B| denotes the size of the mini-batch, while $C^{k}$ refers to the generated sequence for the *k*-th sample. In our specific implementation, we use stochastic gradient descent to minimize our overall loss, with the updated equation as follows:

(13) $$\theta =\theta -\alpha \nabla L_{RL}$$

where α is the learning rate, and $\nabla L_{RL}$ denotes the gradient of the overall loss $L_{RL}$ with respect to the model parameters θ.

The baseline policy gradient algorithm allows the reinforcement learning model to be trained effectively, enabling it to generate the desired output sequences. The primary objective of the reward function is to direct the model toward producing the expected sequences. Meanwhile, the baseline reward plays a crucial role in minimizing the variance in gradient estimates, which contributes to enhancing both the efficiency and stability of the model’s training process.

## 4. Experiments

In this section, we describe the experiments related to event temporal relation extraction. Our experiments aim to verify (1) whether the dependency-based generative approach can provide richer training signals for the model, and (2) whether the baseline policy gradient algorithm can improve the model’s training efficiency and stability. Firstly, we introduce the datasets and evaluation metrics used in this experiment in Section 4.1. Then, we provide detailed experimental settings in Section 4.2. Next, we discuss the main experimental results in Section 4.3. Finally, we present detailed ablation studies and case studies in Section 4.4 and Section 4.5, respectively.

### 4.1. Datasets and Evaluation Metrics

**Datasets**: We conducted experiments on two public benchmarks for event temporal relation extraction, MATRES [51] and TB-DENSE [52], both of which are available for research purposes. TB-DENSE is a densely annotated dataset focused on the most prominent events. It includes six temporal relations— Before, After, Includes, Is_Included, Simultaneous and Vague. MATRES follows a new annotation scheme that focuses on the main timeline, annotating temporal relations between events based only on their starting points, reducing them to four types—Before, After, Equal and Vague. For compatibility and comparison, we used the same data splits as in the previous works [34,53] for the datasets considered. Table 1 provides the statistical details of the two datasets, while Table 2 illustrates the distribution of labels.

**Evaluation Metrics**: To ensure a fair comparison with the previous studies, we adopt the same evaluation metrics as used in [11,37]. On both datasets, we exclude the *Vague* label and calculate the micro-F1 score for all other labels. Specifically, in the evaluation phase, we exclude all ground truth pairs that exhibit *Vague* temporal relations.

### 4.2. Experimental Setup

**Parameters Setting**: In this work, we use the base version of T5 [24] as our generative model. The model parameters are optimized using the AdamW optimizer, and the learning rate is linearly decayed to zero without a warm-up. All the experiments are conducted on Nvidia A40 GPUs, sourced from Nvidia, Santa Clara, California, United States. We use the grid search algorithm to search for all the task hyperparameters based on the micro-F1 score of the development set. The gradients are computed using backpropagation. To ensure reproducibility, we set the random seed to 1741 for all the experiments. The search parameters for MATRES and TB-DENSE are shown in Table 3.

**Comparison Methods**: We compare our proposed generative model with the following baselines:

- Deep Structured [34]: A neural network model that utilizes a structured support vector machine as a scoring function to learn temporal constraints and contextual embeddings.
- Relative Event Time [36]: This model combines an auxiliary task to extract relative time on an event timeline.
- UAST [54]: A self-training framework that takes model uncertainty into account and is specifically designed to quantify it.
- CTRL-PG [55]: A method that uses probabilistic soft logic regularization and global inference at the document level.
- FaithTRE [44]: A model that applies Dirichlet priors before estimating correctness likelihood, with temperature scaling also used to recalibrate model confidence measures after bias mitigation.
- LSTM + knowledge [53]: This model integrates knowledge features learned from external resources and optimizes global consistency through integer linear programming.
- Joint Constrainted Learning [14]: A framework that combines logical constraints and common-sense knowledge for joint training of temporal and sub-event relation extraction.
- HGRU + Knowledge [35]: A neural architecture that handles temporal relations using hyperbolic recurrent units.
- Bayesian-Trans [45]: A Bayesian learning-based approach that models temporal relations as latent variables to integrate external knowledge for event temporal relation extraction.

### 4.3. Main Results

We first compare our model with the latest event temporal relation classification baselines, including methods with or without common-sense knowledge injection. Table 4 reports the overall performance of our model and the baseline methods on MATRES and TB-DENSE. From the table, we can observe the following:

(1) Our proposed method achieves better micro-F1 performance compared to the existing methods on both the MATRES and TB-DENSE datasets. Specifically, our method improves the micro-F1 score by 0.8% over the previous SOTA model on MATRES and by 0.6% on TB-DENSE. This indicates that the T5-based generative approach is more effective than the traditional classification-based methods.

(2) Compared to the previous knowledge fusion methods, our proposed approach shows a significant performance improvement, achieving a 0.8% micro-F1 increase over the SOTA models on both datasets. We observe that incorporating additional training resources generally enhances model performance, surpassing the baseline models. However, our proposed generative model achieves accurate event temporal relation detection results without the need for auxiliary external knowledge.

(3) Additionally, compared to all the existing baselines that use BERT-Base or RoBERTa-Base as the backbone, our method shows more pronounced improvements. On the MATRES dataset, our method improves by 1.8% and 1.3% over BERT-Base and RoBERTa-Base, respectively. On the TB-DENSE dataset, the improvements are 2.8% and 3.3%, respectively. The consistent and stable improvements indicate that our generative method helps the model understand temporal relations more reliably.

(4) Finally, we conduct a comparative analysis of the model’s parameter scale. Our GenTRE model uses the T5-Base architecture with a parameter size of 222.90 M, nearly doubling the parameter scale compared to Base-level models, which allows it to more effectively model complex event temporal relations. At the same time, GenTRE is more lightweight compared to Large-level models, reducing computational overhead while maintaining high performance, thereby achieving a good balance between model complexity and computational efficiency. On the MATRES and TB-DENSE tasks, GenTRE achieved the best micro-F1 scores, even though the Large-level models have a larger parameter size. This indicates that GenTRE, with relatively fewer parameters, demonstrates higher data utilization efficiency and achieves excellent results without relying on additional knowledge enhancement.

### 4.4. Ablation Study

To investigate the contribution of each component, we conduct ablation studies with different settings. Specifically, GenTRE involves three main components, namely maximum likelihood pre-training, dependency path generation, and the baseline policy gradient algorithm. Different experimental settings are indicated by subscripts. Table 5 shows the performance of ablation models on the MATRES and TB-DENSE test sets when removing components from GenTRE. From the table, we can observe the following:

(1) As shown in rows 2, 3, and 4, GenTRE outperforms all these methods in terms of micro-F1 score. To achieve optimal performance, maximum likelihood pre-training, dependency path generation, and the baseline policy gradient algorithm are all crucial. This shows that every component is both essential and justifiable for the proposed model.

(2) In row 2, without the maximum likelihood pre-training, GenTRE’s micro-F1 scores dropped by 2.9% and 3.8% on the two datasets, respectively. This finding suggests that pre-training using maximum likelihood effectively narrows the vast action space in text generation, which in turn enhances the performance of the baseline policy gradient algorithm.

(3) In row 3, we excluded the dependency paths from the output sequence *O*, effectively removing the multi-task learning with dependency path generation. As seen in the table, removing the dependency paths significantly reduces GenTRE’s performance, providing strong evidence for the effectiveness of the multi-task learning strategy based on these paths.

(4) Row 4 shows that after removing the baseline policy gradient algorithm, GenTRE’s micro-F1 scores decreased by 2.3% and 3.1% on the two datasets, respectively. The experimental results show that the baseline policy gradient algorithm effectively reduces the variance in the policy gradient algorithm, which not only improves the stability of the generative model but also enhances its training efficiency.

(5) In rows 5 and 6, the performance of T5, when trained solely with the maximum likelihood objective, is presented. The notably inferior results from this training approach suggest that the integration of the dependency path generation with the baseline policy gradient algorithm yields superior performance in extracting high-quality event temporal relations.

### 4.5. Case Study

To further validate the effectiveness of our method, we analyze examples from MATRES that were successfully predicted by GenTRE but were incorrectly identified by the MLE-trained model (i.e., the model trained only with the maximum likelihood objective). Table 6 provides two cases that illustrate the effectiveness of GenTRE and reveal issues with MLE training. Each case includes a pair of events, and the findings can be categorized into different types. In Case 1, the MLE-trained model generated an incorrect dependency path, missing important contextual words (“others”). In Case 2, the MLE-trained model produced irrelevant/noisy words (“talk” and “gulf”). Such missing or irrelevant information indicates that MLE training fails to encode the essential context for successful temporal relation label prediction.

In summary, our analysis reveals that GenTRE successfully generates the correct dependency paths between event mentions, demonstrating its capability to learn the context needed for precise predictions. Additionally, it has been demonstrated that the baseline policy gradient algorithm effectively mitigates the high variance in gradient estimates, which in turn enhances the model’s performance in predicting temporal relations. In contrast, MLE training often results in incorrect dependency paths, exhibiting limited representational learning ability and leading to failures in temporal relation prediction.

## 5. Analysis and Discussion

As mentioned in the introduction, to further explore the effectiveness of our model, we conducted a series of comprehensive evaluations, including examining the impact of training data size, the effect of reward function weights, and the effectiveness of baseline strategies. We will detail each experiment below, which can provide further insights into the strong performance of our model.

### 5.1. Effect of Training Data Size

**Figure 2:**
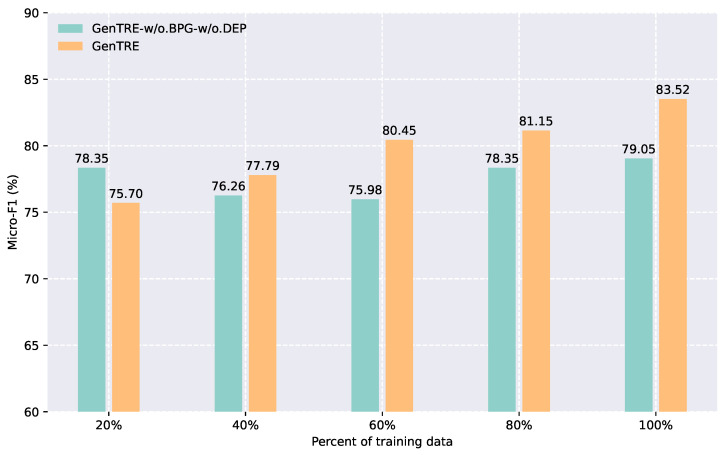
Model performance under different training data sizes (MATRES dataset).

To evaluate the performance of the proposed model under low-resource conditions, we conducted experiments with GenTRE and $GenTRE_{-w/oBPG-w/oDEP}$ on varying amounts of training data. The results are shown in Figure 2. From the figure, we can observe that, as the amount of training data increases, the performance of the GenTRE model steadily improves. This indicates that the scale of the training data has a significant impact on our model, demonstrating that GenTRE has strong learning and generalization capabilities, while also further validating the enhanced effects of dependency path generation and the baseline policy gradient algorithm.

Additionally, we observed that the accuracy of the $GenTRE_{-w/oBPG-w/oDEP}$ model initially showed a higher level but then plateaued. In contrast, the GenTRE model exhibited a consistently increasing trend. This suggests that GenTRE’s training process is more robust, effectively bypassing issues such as local optima and optimization challenges. Such stability enhances the model’s ability to fine-tune its parameters, leading to a gradual improvement in accuracy. It is also noteworthy that the proposed GenTRE model performed less accurately with only 20% of the training data. We hypothesize that the $GenTRE_{-w/oBPG-w/oDEP}$ model may be better suited for specific data characteristics, which explains its higher accuracy on the validation set.

### 5.2. Effect of Reward Function Weights

**Figure 3:**
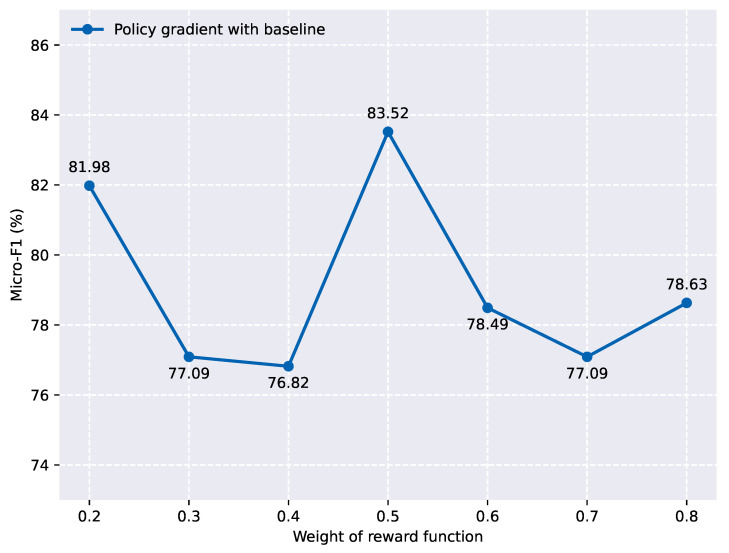
Model performance under different reward function weights (MATRES dataset).

To explore the significance of temporal relation labels and key contextual words in event temporal relation extraction, we assigned varying weights to the reward function in the baseline policy gradient algorithm. The results of this experiment are illustrated in Figure 3, which shows how the micro-F1 score fluctuates as the reward function weights are adjusted. From the figure, it is evident that the model achieves the best performance when the reward function weight is set to 0.5. Any increase or decrease in the weight results in a noticeable decline in performance. This suggests that there is an optimal balance in the weight of the reward function, and deviations from this balance can hinder the model’s ability to effectively capture and utilize temporal relations.

We argue that when the weight of the reward function exceeds 0.5, the reward signal becomes disproportionately dominant, leading the model to become overly sensitive to minor fluctuations in the reward. This excessive sensitivity can prevent the model from adequately considering other important feature information between events, which ultimately compromises the robustness and stability of the learned strategy. On the other hand, if the reward function weight is set below 0.5, the model may downplay the reward signal, causing it to place less emphasis on temporal relation labels during training. This reduction in focus on these labels could impair the model’s ability to accurately capture and model the temporal relationships between events.

### 5.3. Effect of Policy Gradient with Baseline

**Figure 4:**
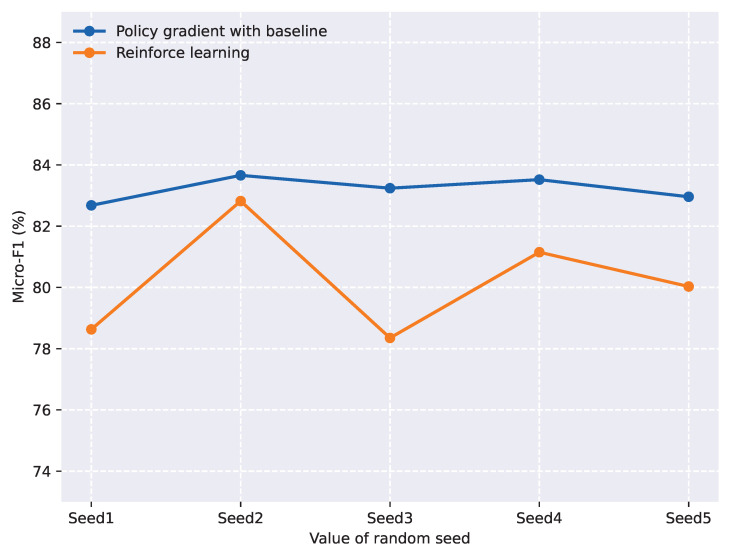
Model performance under five different random seeds (MATRES dataset).

To further validate the effectiveness of the baseline policy, we compared the proposed baseline policy gradient algorithm with a reinforcement learning algorithm. We conducted experiments with five different random seeds under the same model parameter settings, and the results are shown in Figure 4. From the figure, we can observe that the reinforcement learning algorithm exhibited significant overall fluctuation, which greatly affected the model’s prediction results. In contrast, the policy gradient algorithm with a baseline function demonstrated relatively stable micro-F1 scores across the five experiments, averaging around 83. This indicates that incorporating a baseline function into the policy gradient algorithm reduces variance, thereby improving the stability and efficiency of the algorithm.

We chose to compare the baseline policy gradient algorithm with REINFORCE, which is one of the most fundamental and widely recognized policy gradient methods in reinforcement learning. This is because a large number of contemporary policy-based algorithms are derived from REINFORCE, making it a key reference point for comparison. Furthermore, the baseline policy gradient algorithm proposed in this paper is also a variant of policy gradient methods, which share the same foundational principles as REINFORCE. As a result, it is both logical and relevant to compare the two approaches to understand their relative performance and efficacy.

## 6. Conclusions

In this paper, we proposed a reinforcement learning-based generative approach for event temporal relation extraction to address the limitations of the previous methods. Unlike traditional event temporal relation extraction methods, our approach utilizes a generative framework with T5. We explored multi-task learning, using the generative model to produce temporal relations from input sentences and employing dependency path generation as a supplementary task to enhance temporal relation prediction performance. Additionally, we introduced a baseline policy gradient algorithm to address the high variance issue in generative model training. Experiments on two widely used datasets, MATRES and TB-DENSE, demonstrated that our method achieved competitive performance. Thus, the multi-task learning strategy and baseline policy gradient algorithm proposed in this study are practical solutions for addressing issues in existing event temporal relation extraction models. This method introduces a fresh perspective for event temporal relation extraction tasks and has the potential to be adapted for other natural language processing challenges. In future work, enhancing the model’s performance can be achieved by integrating various advanced techniques, as well as exploring its wider applicability. For instance, introducing additional auxiliary tasks could help boost the model’s capabilities, and incorporating technologies like attention mechanisms might improve its generative performance.

## 7. Limitations

Firstly, our method introduces dependency path generation as a supplementary task, which can output the important contextual words needed to predict temporal relations between two event mentions, resulting in higher accuracy. However, dependency paths can include noise and unnecessary sentence components, especially when the two events are in different sentences. Exploring new syntactic strategies to optimize dependency path generation is a challenge. Additionally, the sequence-to-sequence approach suffers from exposure bias [47], which can lead to error accumulation during testing and result in poor performance. Therefore, addressing the mismatch between training and testing phases may yield better outcomes. Lastly, training our model requires substantial GPU resources, which might have an environmental impact, although the inference phase is less resource-intensive.

## Figures and Tables

**Table 1:** Statistical data for MATRES and TB-DENSE datasets.

Dataset	Statistic	Train	Valid	Test
MATRES	Documents	260	21	20
	Event Pairs	10,888	1852	837
TB-DENSE	Documents	22	5	9
	Event Pairs	4032	629	1427

**Table 2:** Label distribution for MATRES and TB-DENSE datasets.

Labels	MATRES	TB-DENSE
Before	427	384
After	271	274
Includes	-	56
Is_Included	-	53
Equal/Simultaneous	30	22
Vague	109	638
Total	837	1427

**Table 3:** Hyperparameters used for MATRES and TB-DENSE.

Stage	Parameters	MATRES	TB-DENSE
Maximum Likelihood Training	Batch Size	32	32
	Learning Rate	0.001	0.001
	Epoch	15	15
	$\alpha_{\mathrm{gen}}$	0.50	0.90
	$\alpha_{\mathrm{re}}$	0.50	0.10
Policy Gradient with Baseline	Batch Size	16	16
	Learning Rate	0.0005	0.0005
	Epoch	15	15
	$\alpha_{\mathrm{acc}}$	0.50	0.75
	$\alpha_{\mathrm{out}\;}$	0.40	0.10
	$\alpha_{\mathrm{in}\;}$	0.10	0.15

**Table 4:** Micro-F1 scores on MATRES and TB-DENSE. Models marked with * use additional training resources. Bold text indicates the optimal result.

Model	Architecture	Params (M)	MATRES	TB-DENSE
LSTM + knowledge *	Bi-LSTM	0.2877	76.3	-
Deep Structured	Bi-LSTM	0.3072	81.7	62.5
CTRL-PG	BERT-Base	109.49	-	65.2
HGRU + knowledge *	RoBERTa-Base	125.01	80.5	-
Bayesian-Trans	RoBERTa-Base	-	82.2	63.0
Joint Constrainted Learning *	RoBERTa-Large	359.56	78.8	-
UAST	RoBERTa-Large	-	80.5	64.3
Relative Event Time	RoBERTa-Large	360.62	81.7	63.2
FaithTRE	BigBird-Large	364.57	82.7	-
Bayesian-Trans *	COMET-BART	204.82	82.7	65.0
GenTRE (Ours)	T5-Base	222.90	83.5	65.8

**Table 5:** An ablation study conducted on the MATRES and TB-DENSE datasets. Bold text indicates the optimal result.

Line	Model	MATRES	TB-DENSE
1	GenTRE (full)	83.5	65.8
2	GenTRE w/o MLE pre-training	80.6	61.9
3	GenTRE w/o dep path	75.7	53.2
4	GenTRE w/o baseline policy gradient	81.2	62.7
5	Only MLE training	78.4	56.6
6	Only MLE training w/o dep path	74.6	54.0

**Table 6:** Examples of successful temporal label generation from GenTRE and incorrect generation from MLE training. Bold text indicates the event pairs in the paragraph.

Input Sentence	GenTRE	MLE Train
The FAA on Friday announced it will close 149 regional airport control towers because of forced spending cuts—**sparing** 40 others that the FAA had been **expected** to shutter.	After. sparing happened after expected because sparing—others—expected.	Before. sparing happened before expected because sparing—expected.
The gulf between the pricing talk from some insurers and the government projections **suggests** how complicated the law’s effects will be. Carriers will be **filing** proposed prices with regulators over the next few months.	Before. suggests happened before filing because suggests—filing.	After. suggests happened after filing because suggests—talk—gulf—filing.

## Data Availability

Public data warehouse. MATRES dataset: https://github.com/qiangning/MATRES (accessed on 15 April 2024). TB-DENSE dataset: https://github.com/muk343/TimeBank-dense (accessed on 15 April 2024).
